# The chloroplast genome of *Salix floderusii* and characterization of chloroplast regulatory elements

**DOI:** 10.3389/fpls.2022.987443

**Published:** 2022-08-26

**Authors:** Weichao Ren, Zhehui Jiang, Meiqi Zhang, Lingyang Kong, Houliang Zhang, Yunwei Liu, Qifeng Fu, Wei Ma

**Affiliations:** ^1^School of Pharmacy, Heilongjiang University of Chinese Medicine, Harbin, China; ^2^School of Forestry, Northeast Forestry University, Harbin, China; ^3^Yichun Branch of Heilongjiang Academy of Forestry, Yichun, China; ^4^Experimental Teaching and Training Center, Heilongjiang University of Chinese Medicine, Harbin, China

**Keywords:** *Salix floderusii*, chloroplast genome, phylogenetic relationship, chloroplast regulatory elements, prokaryotic expression

## Abstract

*Salix floderusii* is a rare alpine tree species in the *Salix* genus. Unfortunately, no extensive germplasm identification, molecular phylogeny, and chloroplast genomics of this plant have been conducted. We sequenced the chloroplast (cp) genome of *S. floderusii* for the first time using second-generation sequencing technology. The cp genome was 155,540 bp long, including a large single-copy region (LSC, 84,401 bp), a small single-copy region (SSC, 16,221 bp), and inverted repeat regions (IR, 54,918 bp). A total of 131 genes were identified, including 86 protein genes, 37 tRNA genes, and 8 rRNA genes. The *S. floderusii* cp genome contains 1 complement repeat, 24 forward repeats, 17 palindromic repeats, and 7 reverse repeats. Analysis of the IR borders showed that the IRa and IRb regions of *S. floderusii* and *Salix caprea* were shorter than those of *Salix cinerea*, which may affect plastome evolution. Furthermore, four highly variable regions were found, including the *rpl22* coding region, *psbM/trnD-GUC* non-coding region, *petA/psbJ* non-coding region, and *ycf1* coding region. These high variable regions can be used as candidate molecular markers and as a reference for identifying future *Salix* species. In addition, phylogenetic analysis indicated that the cp genome of *S. floderusii* is sister to *Salix cupularis* and belongs to the Subgenus *Vetrix*. Genes (Sf-*trnI*, Sf-*PpsbA*, *aadA*, Sf-*TpsbA*, Sf-*trnA*) obtained via cloning were inserted into the pBluescript II SK (+) to yield the cp expression vectors, which harbored the selectable marker gene *aadA*. The results of a spectinomycin resistance test indicated that the cp expression vector had been successfully constructed. Moreover, the *aadA* gene was efficiently expressed under the regulation of predicted regulatory elements. The present study provides a solid foundation for establishing subsequent *S. floderusii* cp transformation systems and developing strategies for the genetic improvement of *S. floderusii.*

## Introduction

*Salix floderusii* (Nakai) is a perennial shrub and member of the genus of *Salix* in the family Salicaceae. *S. floderusii* can reach a height of 4–6 m and grow in rocky areas near the top of mountains, 1,000–1,500 m above sea level. *S. floderusii* is mainly distributed in northeast China, Korea, and the Russian Far East. As an alpine tree species, the unique living environment of *S. floderusii* may influence its morphological characteristics and species evolution. The flora reflects the composition, distribution, origin and evolutionary history of all plant species in a particular area (Zhengyi et al., 2003). The distribution of *S. floderusii* indicates that this area belongs to Asian-North American-Arctic vegetation type (Dong et al., 2017). *S. floderusii* also has a wide range of applications and can be used in painting, baking, and as raw material (Pan et al., 2021).

The chloroplast (cp) genome consists of genetic material unique to plant species. It carries unique genetic information and has its own genetic system composed of a closed circular DNA molecule. The cp genome has a quadripartite structure comprising small single-copy (SSC) and large single-copy (LSC) regions separated by two inverted repeats (Wicke et al., 2011). In recent years, cp genomes have become a better and more popular alternative approach for species identification and phylogenetic analysis of plant relatives as they are more conserved than nuclear genomes (Jansen and Ruhlman, 2012; Yagi and Shiina, 2014; Kim et al., 2015; Park et al., 2018). With the development of high-throughput sequencing technology and low sequencing cost, increasing amounts of cp genomic data have been accumulated (Hatmaker et al., 2020; Shidhi et al., 2021; Wei et al., 2021; Zhang et al., 2021). To date, the cp genome of *Salix* has been reported (Chen et al., 2019; Guo et al., 2021; Li et al., 2021; Gulyaev et al., 2022; Wang et al., 2022). The published cp genomic data of these species offer important references for related research (Lauron-Moreau et al., 2015; Zong et al., 2019; Meucci et al., 2021).

The cp is an important site for photosynthesis in plants and a potential tool for plant genetic transformation. Plastid gene engineering has attracted wide attention because of its advantages, including high expression levels of foreign genes (De Cosa et al., 2001), the absence of position effect and gene silencing (Lee et al., 2003; Dhingra et al., 2004), and the avoidance of environmental safety problems caused by pollen escape in nuclear transformation systems (Svab and Maliga, 2007). Plastid genetic transformation technology has been used to introduce herbicide resistance (Daniell et al., 1998), disease resistance (Ruhlman et al., 2014), insect resistance (De Cosa et al., 2001), and other related exogenous genes into many herbaceous plants (Okuzaki et al., 2013; Chen et al., 2014). However, studies on cp transformation systems in woody plants remain limited. Therefore, the in-depth study of cp transformation in woody plants would explore endogenous regulatory elements further, establish a more efficient cp transformation system, and provide a theoretical basis for improving and breeding new varieties of woody plants.

Currently, the taxonomic status of *S. floderusii* in the genus *Salix* is only at the morphological level. Therefore, it is necessary to clarify the taxonomic status of *S. floderusii* by cp genome at the molecular level and determine the phylogenetic relationship and evolutionary relatedness with related species. In the present study, we sequenced the cp genome of *S. floderusii* and analyzed its structural characteristics. In addition, we constructed an evolutionary tree from the cp genome to explore the evolutionary relationship between *S. floderusii* and other *Salix* plants. Next, a series of cp regulatory elements were cloned from *S. floderusii*. A cp expression vector was constructed, and the biological functions of the regulatory elements were verified via a prokaryotic expression system. Our findings provide a basis for establishing subsequent cp genetic transformation systems and for the study of biology and evolution.

## Materials and methods

### Sample collection and DNA extraction

Mature fresh leaves of *S. floderusii* were obtained from Fenghuang Mountain National Forest Park in the Heilongjiang Province of China (44.107° N, 128.049° E; 1,730 m above sea level). Fresh samples were stored at −80°C for long-term. The voucher specimen was stored at the Institute of Traditional Chinese Medicine, Heilongjiang University of Chinese Medicine. Total genomic DNA was extracted using the Plant DNA Extraction Kit (SIMGEN, Hangzhou, China) according to the manufacturer’s instructions. DNA purity and integrity were verified via 1% agarose gel electrophoresis. Total plant DNA that met the quality requirements was sent to Novogene Bioinformatics Technology Co., Ltd. (Beijing, China) for sequencing using the Illumina high-throughput sequencing platform NovaSeq 6000 via the double terminal sequencing method (150 pair-ends).

### Data filter connected and annotation

Raw data were filtered using the FastQC version 0.11.9 software under the following parameters: (1) remove sequences with N content exceeding 10% of the read length base number, (2) the number of low-quality bases exceeds 50% of the number of bases in the sequence, and (3) retain sequences with unremoved joints to obtain high-quality clean data. The complete cp genome was assembled using the GetOrganelle (Jin et al., 2020) version 1.7.5 software^1^. The GeSeq^2^ (Tillich et al., 2017) software was used to check the *S. floderusii* cp genome sequences after splicing. The online tool CPGAVAS2^3^ (Shi et al., 2019) was used to annotate the cp genomes of *S. floderusii* and *Salix suchowensis* (MT551163) as reference sequences with default parameters. The annotated results were submitted to GenBank (GenBank accession number: OK375876). The file containing the sequence annotations was uploaded to the online drawing software OGDRAW^4^ (Wyman et al., 2004) to generate the cp genome map.

### Cp genome analyses

The CDS sequences were extracted *S. floderusii* cp genome sequence. The relative synonymous codon usage (RSCU) values and codon usage were performed using CodonW version 1.4.2 with default settings (Wong et al., 2010). The MISA (Beier et al., 2017) on-line analysis software^5^ was used to analyze simple sequence repeat (SSR) of the cp genome. The thresholds used to detect the SSRs were 8,4,4,3,3,3 for mono-nucleotide, di- nucleotide, tri-nucleotide, tetra- nucleotide, penta- nucleotide, and hexa-nucleotides, respectively. The shortest size between any two SSR loci was more than 100 bp. The online software REPuter^6^ (Kurtz et al., 2001) was carried out on the *S. floderusii* of cp genome repeat sequence analysis. The analysis included forward, reverse, complement, and palindromic repeats under the following parameters: maximum computed repeats 50; hamming short 3; minimal repeat size of 20 bp; default values for other parameters.

### Comparative sequence analysis

*Salix* sect. *Vetrix* species were analyzed for genome expansion and shrinkage using the online software IRscope^7^ (Amiryousefi et al., 2018). Cp genome of three species collinearity analysis was performed using the MAUVE version 2.4.0 (Darling et al., 2004) software. Additionally, the mVISTA^8^ (Frazer et al., 2004) online analysis software was used under the shuffle-Lagan analysis mode, with *p* = 0.5 and *S. caprea* used as a reference. The cp genome sequence diversity of the three species of *Salix* Sect. *Vetrix* was analyzed using the DnaSP version 6.12.03 (Rozas et al., 2017) software. The π value of plastids was calculated through the sliding window. The parameters under the sliding window were set as follows: window length = 600 bp; step size = 200 bp.

### Phylogenetic analysis

Phylogenetic analysis was conducted on cp genomes of 45 *Salix* species, using *Populus davidiana* as an outgroup. Details of the species, downloaded from NCBI, are shown in Supplementary Table 1. First, the cp genomes of the 46 species were aligned using the Mafft version 7.313 software, based on default parameters. Next, a maximum-likelihood (ML) phylogenetic tree was constructed via IQ-TREE version 1.6.8 by selecting the K3Pu + F + G4 computational model with 1,000 bootstraps by PhyloSuite version 1.2.2 (Zhang et al., 2018) analysis software. Bayesian inference (BI) analysis was performed using the Markov Chain Monte Carlo (MCMC) algorithm in MrBayes version 3.1.27 (Ronquist and Huelsenbeck, 2003). The MCMC analysis was run for 1,000,000 generations independently, using a sampling frequency of 100. The initial 25% of trees were discarded as burn-in, and the remaining data were used to construct a majority-rule consensus tree and default values for other parameters. Finally, the evolutionary tree was visualized and refined using MEGA X (Kumar et al., 2018).

### Gene cloning of Cp regulatory elements

The Sf-*PpsbA* fragment contained the *S. floderusii psbA* promoter and the *S. floderusii psbA* leader. Sf-*TpsbA* was the terminator of the *psbA* gene. The Sf-*trnI* or Sf-*trnA* fragment contained the *trnI* or *trnA* gene, respectively, as well as part of the flanking sequence of this gene. The target genes (Sf-*trnI*, Sf-*PpsbA*, Sf-*TpsbA*, and Sf-*trnA*) and a selectable marker gene aadA were cloned using high-fidelity DNA polymerase KOD-Plus (Toyobo, Osaka, Japan). Primer sequences are shown in Supplementary Table 2. Sequence amplification procedures were as follows: pre-denaturation at 98°C for 30 s, followed by 35 cycles of denaturation at 98°C for 10 s, annealing at 58°C for 30 s, and extension at 72°C for 1 min. The target fragment was recycled at 72°C for 7 min and used to construct the cp expression vector.

### Construction of recombinant plasmid

We constructed a cp transformation operon structure based on the pBluescript II SK (+) no-load plasmid using the Hieff Clone^®^ Universal One Step Cloning Kit (YEASEN, China). In the process of primer involvement, we followed the homologous arm consisting of a 15–25 bp overlap region at both ends of the target gene and the linearized vector for recombination. All the target segments required to construct the cp expression vector were assembled into synthetic operons, as described below.

During the first round of target gene construction, *Xho*I and Hind III restriction endonucleases were selected for the enzymatic digestion of pBluescript II SK (+) no-load plasmids to obtain linearized vectors. The Sf-*PpsbA*, *aadA*, and Sf-*TpsbA* gene fragments were assembled into the linearized vector to synthesize pB + aadA using seamless connection kit.

During the second round of target gene construction, the *Kpn*I restriction endonuclease was selected to digest the recombinant plasmid (pB + aadA) —constructed in the previous step—to obtain the linearized vector. pB + Sf-*trnI* + aadA was synthesized from the *trnI* gene fragment and assembled into the linearized vector using a seamless connection kit.

During the third round of target gene construction, the recombinant plasmid (pB + Sf-*trnI* + aadA) constructed in the previous step was digested with the *Bam*HI restriction endonuclease to obtain the linearized vector. pB + Sf-*trnI* + aadA + Sf-*trnA* was synthesized by assembling *trnA* gene fragments with linearized vectors using a seamless connection kit. The steps involved in constructing the cp expression vector are depicted in Supplementary Figure 1.

### Expression analysis of the prokaryotic *aadA* gene

The recombinant plasmid was transformed into *E. coli* strain DH5α. The transformed *Escherichia coli* was incubated in LB liquid medium containing ampicillin (50 ng/μL) and spectinomycin (50 ng/μL) at 37°C and 200 rpm/min for 48 h.

## Results

### Characterization of the Cp genome

**FIGURE 1:**
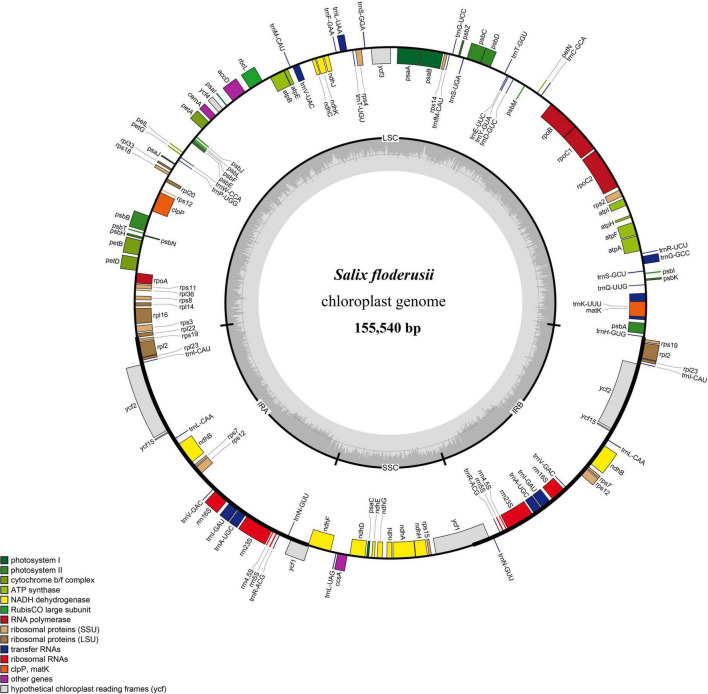
Gene map of the complete *Salix floderusii* cp genome. Genes drawn within the circle are transcribed in a clockwise direction, and genes drawn out are transcribed in a counterclockwise direction.

Similar to most land angiosperms, the cp genome of *S. floderusii* was found to be a closed-loop double-stranded DNA molecule with a typical quadripartite structure. It was 155,540 bp long, including a large single copy (LSC) region of 84,401 bp, a small single copy (SSC) region of 16,221 bp, and a pair of inverted repeats (IRa and IRb) 27,459 bp in length (Figure 1). The GC content in the whole genome was 36.7%, including 41.86% in the inverted repeat (IR) region, 34.45% in the LSC region, and 30.97% in the SSC region. A total of 131 genes were identified, including 86 protein genes, 37 tRNA genes, and 8 rRNA genes. There were 16 genes containing introns in the cp genome of *S. floderusii*, including six tRNA genes (*trnA-UGC*, *trnG-GCC*, *trnI-GAU*, *trnK-UUU*, *trnL-UAA*, and *trnV-UAC*). Ten protein-coding genes (*atpF*, *clpP*, *ndhA*, *ndhB*, *petB*, *petD*, *rpl16*, *rpl2*, *rpoC1*, and *ycf3*) were identified, among which *ycf3* and *clpP* contained two introns (Table 1).

**TABLE 1:** List of genes in the *Salix floderusii* cp genome.

Category of genes	Group of genes	Name of genes
Self replication	tRNA	*trnA-UGC*^a,b^, *trnC-GCA*, *trnD-GUC*, *trnE-UUC*, *trnF-GAA*, *trnfM-CAU*, *trnG-GCC*^b^, *trnG-UCC*, *trnH-GUG*, *trnI-CAU*^a^, *trnI-GAU*^a,b^, *trnK-UUU*^b^, *trnL-CAA*^a^, *trnL-UAA*^b^, *trnL-UAG*, *trnM-CAU*, *trnN-GUU*^a^, *trnP-UGG*, *trnQ-UUG*, *trnR-ACG*^a^, *trnR-UCU*, *trnS-GCU*, *trnS-GGA*, *trnS-UGA*, *trnT-GGU*, *trnT-UGU*, *trnV-GAC*^a^, *trnV-UAC*^b^, *trnW-CCA*, *trnY-GUA*
	rRNA	*rrn4.5*^a^, *rrn5*^a^, *rrn16S*^a^, *rrn23*^a^
	Large subunit of ribosome	*rpl14*, *rpl16*, *rpl2*^a^, *rpl20*, *rpl22*, *rpl23*^a^, *rpl33*, *rpl36*
	DNA dependent RNA polymerase	*rpoA*, *rpoB*, *rpoC1*, *rpoC2*
	Small subunit of ribosome	rps11, rps12^a^, rps14, rps15, rps18, rps19^a^, rps2, rps3, rps4, rps7^a^, rps8
Photosynthesis	ATP synthase	*atpA*, *atpB*, *atpE*, *atpF*, *atpH*, *atpI*
	Photosystem II	*psbA*, *psbB*, *psbC*, *psbD*, *psbE*, *psbF*, *psbI*, *psbJ*, *psbK*, *psbL*, *psbM*, *psbN*, *psbT*, *psbZ*, *ycf3*^c^
	NADH-dehydrogenase	*ndhA*, *ndhB*^a^, *ndhC*, *ndhD*, *ndhE*, *ndhF*, *ndhG*, *ndhH*, *ndhI*, *ndhJ*, *ndhK*
	Cytochrome b/f complex	*petA*, *petB*, *petD*, *petG*, *petL*, *petN*
	Photosystem I	*psaA*, *psaB*, *psaC*, *psaI*, *psaJ*
	Rubisco	*rbcL*
Other genes	Acetyl-CoA-carboxylase	*accD*
	c-type cytochrome synthesis gene	*ccsA*
	Envelop membrane protein	*cemA*
	Protease	*clpP* ^c^
	Maturase	*matK*
Unknown	Conserved open reading frames	*ycf1*^a^, *ycf15*^a^, *ycf2*^a^, *ycf4*

^a^Gene with copies; ^b^genes with one intron; ^c^genes with two introns.

### Codon usage

**FIGURE 2:**
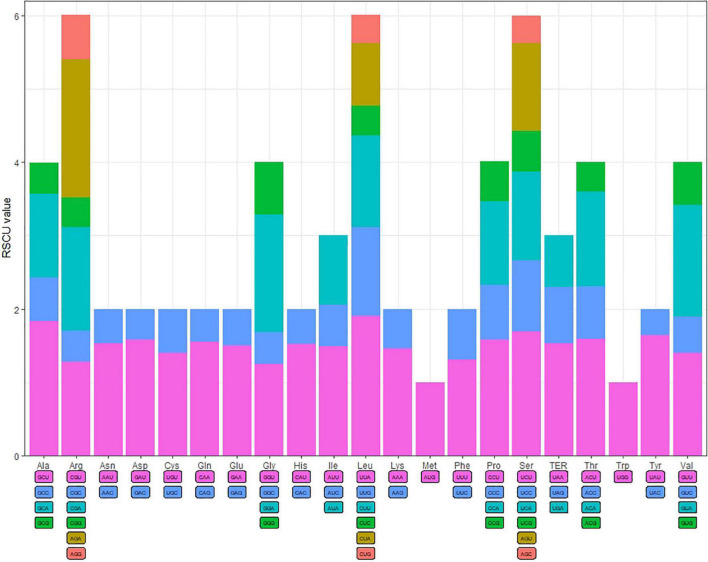
Codon content and codon usage of 20 amino acids and stop codons in the protein-coding genes of the *S. floderusii* cp genome.

The codon usage frequency and RSCU were analyzed based on 86 protein-coding genes in the *S. floderusii* cp genome (Figure 2). A total of 20 amino acids and 26,654 codons were identified. Specifically, AUU (1,149) of isoleucine was the most abundant codon, whereas the least abundant codon was UGC (92) of cysteine. Methionine and tryptophan were encoded by only one codon, and the number of amino acids encoding six codons, four codons, three codons, and two codons was 3, 5, 1, and 9. The highest RSCU value (1.9) corresponded to the UUA codon of Leucine (Leu). The lowest RSCU value was 0.36, corresponding to the UAC codon of Tyrosine (Tyr). Furthermore, 29 codons had RSCU values greater than 1, 30 codons had RSCU values less than 1, and the RSCU values of AUG and UGG were equal to 1.

### Repeat sequence and simple sequence repeat analysis

**FIGURE 3:**
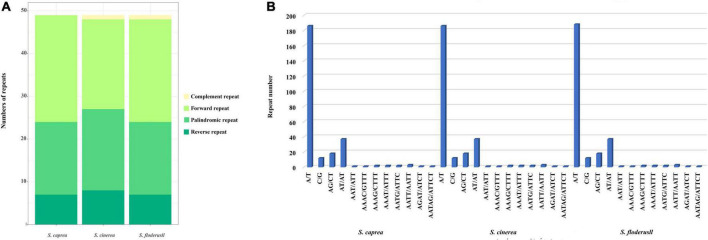
Statistics of simple sequence repeats of the cp genome of *S. floderusii*. **(A)** Number of different repeat types. **(B)** Number of each identified SSRs motif.

We performed repeated sequence analysis on the cp genomes of the three *Salix* species. The number of repeat sequences for each of the three species is 49 (Figure 3A). *S. caprea* contains 25 forward repeats, 17 palindromic repeats, and 7 reverse repeats. *S. cinerea* contains complement repeat one, forward repeat 21, palindromic repeat 19, reverse repeat eight; *S. floderusii* contains 1 complement repeat, 24 forward repeats, 17 palindromic repeats, and 7 reverse repeats. Although the total number of repeat sequences in the three species was the same, the types of repeat sequences were different. *S. cinerea* and *S. floderusii* both contain a complement repeat, unlike *S. caprea*.

Simple sequence repeat analysis was performed on the three cp genomes to identify mononucleotide, dinucleotide, trinucleotide, tetranucleotide, and hexanucleotides repeat motifs, as detailed in Figure 3B. SSRs can be divided into 12 types. A total of 268, 266, and 266 SSRs were detected in *S. floderusii*, *S. caprea*, and *S. cinerea*, respectively. The numbers of SSRs in the three species differed only in A/T type, with 188 for *S. floderusii* and 186 for *S. caprea* and *S. cinerea*. The number of other SSR types in the three species was consistent (Supplementary Table 3). *S. floderusii* contains polythymine (poly T) repeat at 43,872 bp compared with *S. cinerea* and *S. caprea* and *S. cinerea* was deletion polyadenine (polyA) repeat at 117,925 bp. In addition, *S. floderusii* (124,782 bp) and *S. cinerea* (124,813 bp) contain poly T repeat, whereas it was not found in *S. caprea* (Supplementary Table 4).

### Inverted repeat contraction and expansion

**FIGURE 4:**
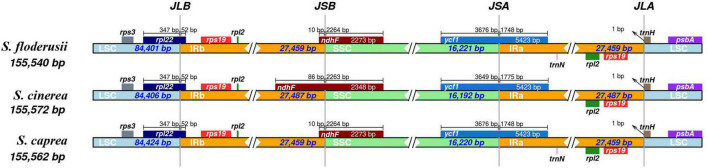
Comparison of the border pattern of large single-copy (LSC) region, small single-copy (SSC) region, and an inverted repeat (IR) region among the *S. floderusii*, *S. cinerea*, and *S. caprea* cp genomes.

The contraction and expansion of the IR region can reflect the evolutionary relationship of species. The cp genome sequences of the three species were very similar in length (155,540–155,572 bp) when compared, and the gene composition and arrangement patterns in each junction region of the three species were roughly equal (Figure 4). The IRa and IRb lengths of *S. floderusii* and *S. caprea* were equal; however, LSC and SSC lengths were slightly varied. The LSC of *S. floderusii* was 23 bp shorter than *S. caprea* and the SSC of *S. floderusii* was 1 bp larger than *S. caprea*. The IRa and IRb regions of *S. floderusii* and *S. caprea* contracted compared with those of *S. cinerea*.

In addition, the sequence of genes at the junction of the LSC and IRb regions was *rps3*, *rpl22*, *rps19*, and *rpl2*. *rpl22* extended by 52 bp to the IRb region. The genes *ndhF* and *ycf1* were located at the junction region of IRb/SSC and SSC/IRa, respectively. The length of the *ndhF* gene and its location at the IRb/SSC junction region were the same in *S. floderusii* and *S. caprea*; however, the *ndhF* gene of *S. cinerea* had the largest extension (86 pb) into the IRb/SSC border. Only *ycf1* was found at the junction of the SSC and IRa. The sequence of genes at the junction of IRa and LSC was *rpl2*, *rps19*, *trnH*, and *psbA*.

### Nucleic acid polymorphism analysis

**FIGURE 5:**
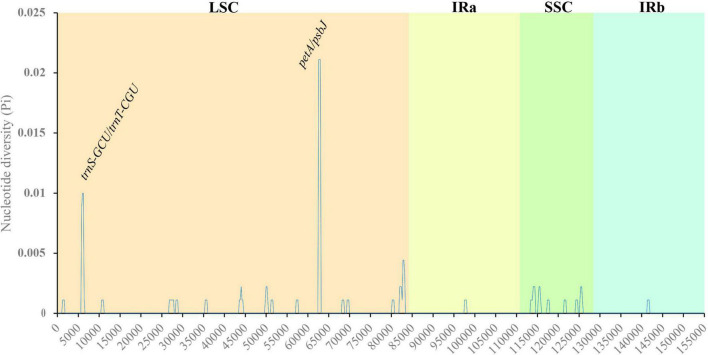
Variation in nucleotide diversity across the cp genomes of the three studied *Salix* species. *X*-axis: position of the midpoint of a window; *Y*-axis: nucleotide diversity of each window.

Nucleotide polymorphism analysis can be used for species identification by comparing the highly variable sequences in related species. Nucleotide polymorphisms of the cp genomes of three Salix species were analyzed, and the results indicated that the Pi value ranged from 0.02111 to 0, with an average value of 0.000262 (Figure 5). Compared with LSC and SSC, the polymorphism of nucleic acids in the IR region was low, indicating that the IR region was more conserved. Pi was greater than 0.005, and we detected two gene spacers, *petA/psbJ* and *trnS-GCU/trnT-CGU*. These nucleotide variation sites can be used as molecular markers for species identification and provide an alternative strategy for species identification.

### Comparative genomic analysis

**FIGURE 6:**
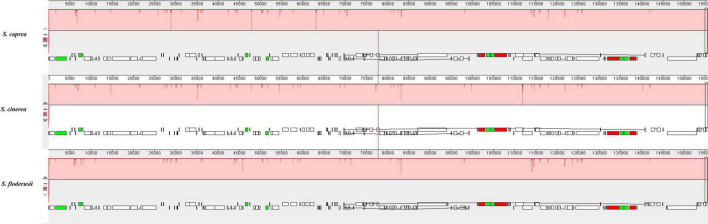
Comparison of the genome structure of three species using the MAUVE program. The DNA sequences above the line are presented in a clockwise direction, and those below the line in a counterclockwise direction.

Comparative genome analysis can identify differences in the gene structure and composition of closely related species more intuitively, as well as distinguish among them. In the present study, we performed MAUVE analysis on *S. cinerea*, *S. caprea*, and *S. floderusii* (Figure 6). The results showed that the entire genome sequence was a homologous region with no big indels. The cp genomes of the three *Salix* species connected with only one locally collinear block, suggesting a high level of similarity, as well as no rearrangement in gene organization.

**FIGURE 7:**
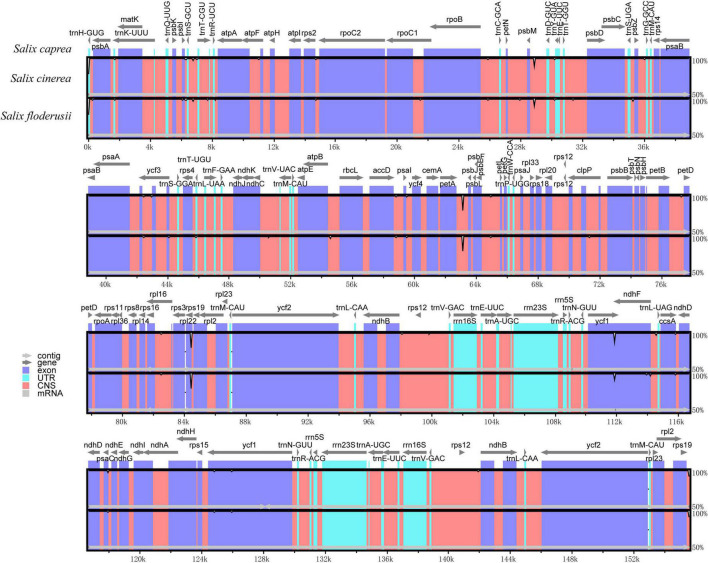
Comparison of three cp genomes using the mVISTA alignment program. Genome regions are color-coded as protein-coding, rRNA coding, tRNA coding, or conserved non-coding sequences. The vertical scale shows the percentage of identity, varying from 50 to 100%.

Analysis using mVISTA showed that the cp genomes of the three *Salix* species were also highly conservative (Figure 7). In addition, the coding region was more conservative than the non-coding region, and the mutation loci mainly appeared in the non-coding region. Comparative analysis of the LSC region, SSC, and IR regions, showed that the LSC region contained more hypervariable regions than the SSC region. By contrast, the IR region had no hypervariable region. The high variable regions include the *rpl22* coding region, *psbM/trnD-GUC* non-coding region, *petA/psbJ* non-coding region, and *ycf1* coding region.

### Phylogenetic analysis

**FIGURE 8:**
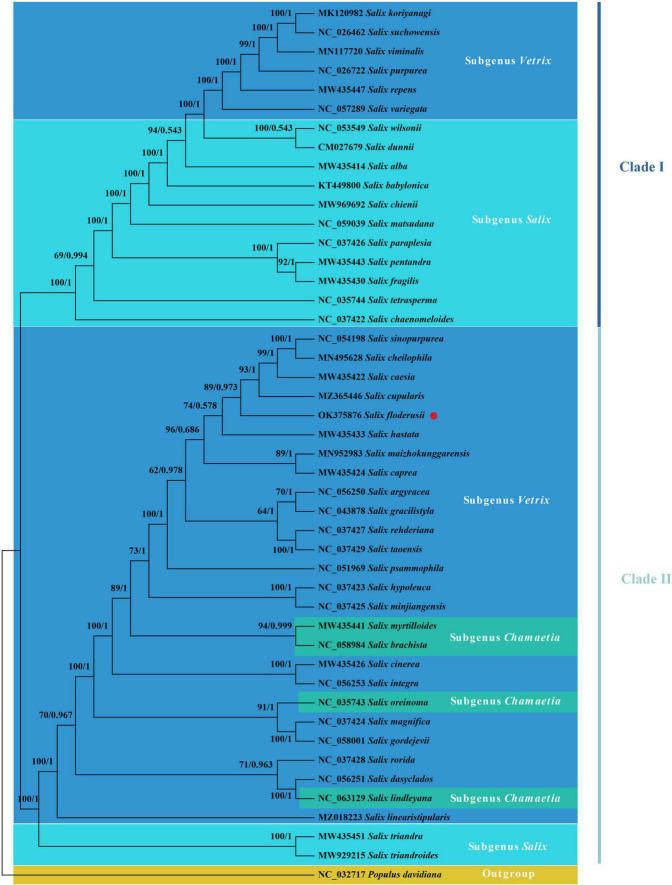
Phylogenetic analysis. The phylogenetic tree inferred from maximum likelihood (ML) and Bayesian inference (BI) of 46 cp genomes from 45 species from the *Salix* genus and *Populus davidiana* as an outgroup. The numbers above the branches are the posterior probability and likelihood values, respectively. The species studied in the present study was marked using red circles.

Phylogenetic trees can accurately describe the evolutionary status of species in their genus. Our findings show that the evolutionary tree of the *Salix* cp genome can be divided into two clades and that all branches have high confidence values (Figure 8). Clade I include Subgenus *Salix* and a few subgenus *Vetrix* species. Subgenus *Salix* includes Sect. *Wilsoniana*, Sect. *Salix*, Sect. *Pentandrae*, and Sect. *Tetraspermae.* The main body of Clade II is Subgenus *Vetrix*, and also includes Sect. *Amygdalinae* of Subgenera *Salix* and *Chamaetia*. *S. floderusii* belongs to Clade II and is closely related to *S. cupularis.*

### Cp regulatory elements in *S. floderusii*

The cloned cp regulatory element sequence can be used for subsequent vector construction. The band length of all amplified sequences was equal to that of the target fragment, as shown in Supplementary Figure 2.

### Analysis of *aadA* gene expression

**FIGURE 9:**
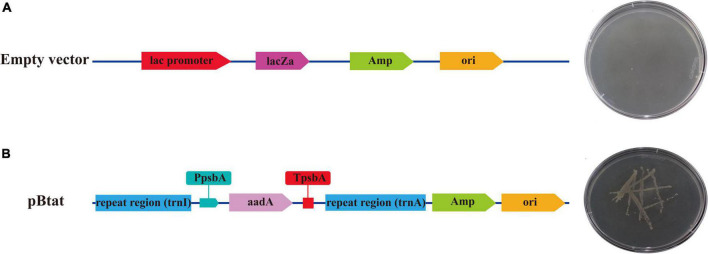
The expression of *aadA* in *Escherichia coli.* **(A)** *Escherichia coli* containing empty vector. **(B)** *Escherichia coli* containing the recombinant vector.

The verified vector was transformed into *E. coli* strain DH5α for expression. *E. coli* containing no load could not grow in LB solid medium containing ampicillin and spectinomycin (Figure 9A). However, recombinant plasmids containing repeat sequences (Sf-*trnI* and Sf-*trnA*), initiating Sf-*PpsbA* and terminating Sf-*TpsbA* and *aadA* genes were able to grow in LB solid media containing ampicillin and spectinomycin (Figure 9B). The results showed that the selected regulatory elements had prokaryotic characteristics and could regulate the correct expression of exogenous genes.

## Discussion

### Highly conserved Cp genomes of *Salix*

Chloroplast genomes have low evolution rate and highly conserved and have thus been found to be ideal in studies of plant phylogeography and molecular evolution, as well as phylogenetic analysis (Burke et al., 2012; Huang et al., 2014). Whereas, inversions, gene or intron losses also occur occasionally (Bell et al., 2010). Although introns are not directly expressed and involved in physiological activities, they can enhance the gene expression level, on the special position, in the specific time (Yi et al., 2012). Ycf3 is required for the stable accumulation of the photosystem I (PS I) and PSII. OTP51 is required for the optimal splicing of several plastid introns, especially ycf3 intron 2, and affect the assembly of PS I and PSII (de Longevialle et al., 2008). The previous studies show that the ClpP gene contains two introns make the low selection effect ratio (ka/ks = 0) of the ClpP gene (Liang and Chen, 2021; Liang et al., 2022). The evolution rate of the ClpP gene is related with species that is clearly evident. The rapid evolution of species will lead to the loss of introns, while it will also be subject to the functional constraints of species lineage (Dugas et al., 2015; Williams et al., 2019).

The genetic information carried on DNA is transmitted as triplet codons during the transfer from RNA to protein. Each amino acid corresponds to an unequal number of codons. In the case of natural selection or mutation preference, the use of codons is biased during protein synthesis. All codons, Ile with a frequency of 8.67% and Cysteine (cys) was the least common one with a proportion of 1.14% (Supplementary Table 5). An interesting phenomenon was found after the codon usage analysis of *S. floderusii*. When the RSCU value was greater than 1, most codons ended in A and U, and only A few codons ended in G. However, when the RSCU value was less than 1, most codons ended with G and C, and only a few codons ended with A. In addition, codon species with RSCU values greater than 1 and less than 1 were very close in number. This phenomenon was also observed in the cp genomes of land plants, such as *Ailanthus altissima* (Saina et al., 2018), *Lycium chinense* (Yang et al., 2019), *Symplocarpus renifolius* (Kim et al., 2019), *Alpinia katsumadai* (Li et al., 2020b). The codon usage frequency was different in cp genome, which might be related to the hydrophilicity, synonymy substitution rate, gene length (Huang et al., 2014), and expression level of the codon, with highly expressed genes displaying higher codon bias (Zhang et al., 2012). The result is closely correlated with the evolutionary pattern of the species (Liu and Xue, 2005).

The SSRs are tandem repeats distributed across the entire genome. In the cp genome, SSRs exhibit high polymorphism, as well as parthenogenetic patterns, and have been widely used in studies investigating population genetics, phylogenetic relationships, gene flow, and pedigree geography (Khadivi-Khub et al., 2013; Li et al., 2020a). In the present study, SSR sequences in the three cp genomes were mainly composed of poly-A and poly-T repeats, contributing to the AT abundance of these genomes. The mononucleotide motif A/T was the most abundant, accounting for 86.19% of the SSRs, and the SSRs were mostly located in non-coding regions. This trend has been demonstrated in several previous studies (Gao et al., 2019; Yan et al., 2019). These SSR markers can be used to develop specific markers and are the key to systematic research.

### Contraction and expansion of inverted repeat regions

The IR region is the most conserved region of the cp genome. The expansion and contraction of IR, LSC, and SSC regions are common phenomena during evolution and the main reason for variations in cp genome length (Daniell et al., 2008; Xue et al., 2019). In the present study, the cp genomes of the three *Salix* Sect. *Vetrix* species were highly conserved at the LSC/IRb, SSC/IRa, and IRa/LSC junction sites, which showed slight differences in the length of partial genes, their location at the junction, and the deletion of the gene. The results of the present study, showed that the *ndhF* gene length and located at the IRb/SSC junction site differed among *S. cinerea*, *S. floderusii*, and *S. caprea*. The length variation of *ndhF* gene of the three *Salix* species is similar to those identified in previous studies reported that the ndhF also shows the highest rate of base substitution in the Asteraceae (Olmstead et al., 2000) and Berberidaceae (Kim et al., 2004). By contrast, the *ycf1* gene located at the SSC/IRa junction site was conserved among the closely related species of the family. Therefore, we inferred that the junction between the LSC and IR regions was relatively conserved, while the junction between the SSC and IR regions was highly variable. Similar results were obtained in most high plants, as reported in previous studies (Tian et al., 2002; Wang et al., 2013). In addition, the *trnN* gene, one of the important genes in the cp, was absent from the IRa region of *S. cinerea*. Studies have shown that the deletion of *trnN* leads to a significant decrease in the abundance of photosystem I core complex, cytochrome b6f complex, photosystem II core complex, and ATP synthase subunit, which damages cp translation, consequently affecting the accumulation of plastid coding proteins (Legen et al., 2007). Thus, *trnN* is essential to the mechanism underlying plastid translation.

### Comparative genomic analysis

Comparative analysis of the cp genomes of three *Salix* species revealed four highly variable regions, namely the *rpl22* coding region, *psbM/trnD-GUC* non-coding region, *petA/psbJ* non-coding region, and *ycf1* coding region. Previous research has showed that *rpl22-rps19-rpl2*, *psbM-trnD*, and two other highly variable regions can be considered the most promising variable factors for species division and in population genetics studies (Wang et al., 2021). It has been reported that six highly variable loci, namely *trnH-GUG/psbA*, *ndhF*, *ndhF/rpl32*, *trl32/trnL-UAG*, *ndhD*, and *ycf1*, can be used as candidate loci for plasmid barcoding for species identification in the phylogenetic analysis of *Lindera* species (Tian et al., 2019). Among medicinal plants, 95 ginseng samples have been screened for DNA barcoding. Moreover, significantly different Panax bipinnatifidus species were identified using *matK*, *psbK-I*, *psbM-trnD*, *rps16*, and *nadI* (Zuo et al., 2010). The *ndhf-rpl32*, *psbM-trnD*, and *trnS-trnG* regions are the most rapidly evolving regions in ornamental plants (Lu et al., 2018), with *psbM-trnD* playing an important role in species identification. *petA-psbJ* may be used as a divergent region to develop potential DNA barcodes to identify *Pyrrosia* species (Yang et al., 2020). Species diagnosis for most of the *Kaempferia* genus was conducted using the loci of *psbA-trnH* and *petA-psbJ* as DNA barcodes (Techaprasan et al., 2010). *petA-psbJ* can be used as a candidate fragment for DNA barcoding in phylogeny and species identification of other species, including those of the *Rosa* (Yin et al., 2020), *Bupleurum* (Xie et al., 2021), and *Zingiber* (Li et al., 2020c) genera, as well as the Papaveraceae (Liu et al., 2020) and Lauraceae (Jo et al., 2019) families. These hypervariable regions are suitable candidates for species identification and provide evolutionary information about *Salix* species.

Comparative analysis of the LSC, SSC, and IR regions, showed that hypervariable regions were mainly distributed in the LSC and SSC regions, with the LSC region containing the highest number of variable regions. By contrast, the IR region contained no highly variable region. The main explanation is that LSC and SSC regions are not conserved, whereas the IR region is more conserved, indicating that the evolution rate of non-coding sequences in *Salix* is faster than that of protein-coding gene sequences.

### Phylogenetic analysis

Phylogenetic analysis of the cp genomes of 45 *Salix* species showed that all species were monophyletic. Several studies have been published on the phylogeny of the genus *Salix* and on phylogenetic analyses that used the protein-coding region of the cp genome, as well as the whole cp genome (Azuma et al., 2000; Lauron-Moreau et al., 2015; Huang et al., 2017). Given the limited number of *Salix* species involved in a previous study (Wu et al., 2015), we compared a higher number of *Salix* species for phylogenetic analysis and aimed to display the phylogenetic status of *Salix* more comprehensively. The two main clades formed within the *Salix* genus were generally consistent, similar to the result obtained by Chen et al. (2019). In the Subgenus *Vetrix*, each species section is not grouped into one but mixed, consistent with Skvortsov’s morphological description of Sect. *Helix* and Sect. *Incubaceae* species. According to Skvortsov, these two species sections have a spiral leaf arrangement, typically seen in the willows (where the divergence angle of 2/5 is retained) (Skvortsov, 1999). The phylogenetic tree showed that most species from the same Section could not cluster into a branch. The main reasons for this phenomenon are as follows: (1) due to the selection of different datasets, the interspecific relationships presented by the phylogenetic tree constructed in this study are slightly inconsistent compared with the results of previous studies; (2) other studies have also shown that *Salix* is not monophyletic, mainly due to its widespread hybridization, which leading to multiple evolving networks and an unstable morphological classification based on the difference of reproductive organs (Wu et al., 2015); (3) warmer climates were shown to alter the increasing relative abundance of *Salix*, which showed higher genetic diversity in arctic ecosystems, highlighting the significant role of environmental factors in the evolution of species (Meucci et al., 2021). Therefore, further studies are needed to clarify the classification and phylogeny of *Salix*.

### Analysis of *aadA* gene expression in the prokaryotic expression vector

Selecting appropriate active promoters with 5′ UTR and 3′ UTR is the first condition to construct prokaryotic expression vectors and establish efficient transformation systems. Generally, using endogenous host genes can markedly facilitate the integration and expression of foreign genes in the host genome (Radakovits et al., 2010). In the present study, two homologous recombinant fragments, one high-efficiency promoter and one 3′ UTR sequence, were identified from the *S. floderusii*. The 3′ UTR sequence usually contains a stable stem-loop structure—only thus can it ensure the stability of mRNA and facilitate the accumulation of proteins in the prokaryotic expression system (Monde et al., 2000). RNA secondary structure prediction analysis of *TpsbA* showed that the gene has a stable stem-loop structure.

The aminoglycoside 3-adenylate transferase encoded by *aadA* can inactivate spectinomycin and streptomycin by adenylate acylation, preventing them from binding to ribosomes. Plants transformed with *aadA* are thus resistant to spectinomycin and streptomycin—a trait that can be used to screen green transformed cells and white non-transformed cells. Svab and Maliga (1993) successfully transformed tobacco cp (Chen et al., 2014; Yarbakht et al., 2015) using the *aadA* gene as a selective marker. To date, this transformation system has been applied in *Arabidopsis thaliana* (Sikdar et al., 1998), potato (Sidorov et al., 1999), soybean (Dufourmantel et al., 2004), rice (Lee et al., 2006), and other herbaceous plants (Liu et al., 2007; Cheng et al., 2010; Wei et al., 2011; Harada et al., 2014; Lelivelt et al., 2014; Muralikrishna et al., 2016). In the present study, the *aadA* gene was selected as an effective selective marker to ensure that the selected regulatory elements can perform their biological functions normally. Because of the high similarity in the transcription and translation systems between *E. coli* and chloroplasts, a combined strategy using both the *E. coli* and chloroplast systems could potentially be applied to develop subsequent chloroplast expression vectors for plant transformation.

## Conclusion

In the present study, we sequenced, assembled, and annotated the cp genome of *S. floderusii* using high-throughput technology and comprehensively analyzed its gene structure. Comparative analysis of the *S. floderusii* cp genome with those of related species showed that the three species in sect. *Vetrix* were conserved in terms of gene structure. We identified the phylogenetic position of *S. floderusii* in the genus *Salix* by constructing a phylogenetic tree. Furthermore, we cloned and identified a series of cp expression elements with characteristics of prokaryotic expression in *S. floderusii*. These findings provide a basis for establishing a cp genome transformation system and are essential for conserving and utilizing *Salix* germplasm resources.

## Data availability statement

The original contributions presented in this study are publicly available. The annotated complete chloroplast genome sequences were submitted to the NCBI database with accession number OK375876, and the original source data can be found here: https://www.jianguoyun.com/p/Dap53uUQ3PDeChj5zcsEIAA.

## Author contributions

YL, QF, and WM designed the experiments. WR, HZ, and ZJ carried out the experiments. WR, MZ, and LK analyzed the experimental results. WR, ZJ, and MZ wrote the manuscript. All of the authors have approved the final manuscript.

## Footnotes

1
https://github.com/kinggerm/getorganelle/blob/master/get_organelle_from_assembly.py

2
https://chlorobox.mpimp-golm.mpg.de/geseq.html

3
http://47.96.249.172:16019/analyzer/home

4
https://chlorobox.mpimp-golm.mpg.de/OGDraw.html

5
http://pgrc.ipk-gatersleben.de/misa/misa.html

6
https://bibiserv.cebitec.uni-bielefeld.de/reputer

7
https://irscope.shinyapps.io/irapp/

8
https://genome.lbl.gov/vista/mvista/submit.shtml
