# Supplementary figures and images for: The chloroplast genome of Salix floderusii and characterization of chloroplast regulatory elements
Source: Front Plant Sci. 2022 Aug 26;13:987443. doi: 10.3389/fpls.2022.987443 (PMC9459086; doi:10.3389/fpls.2022.987443)

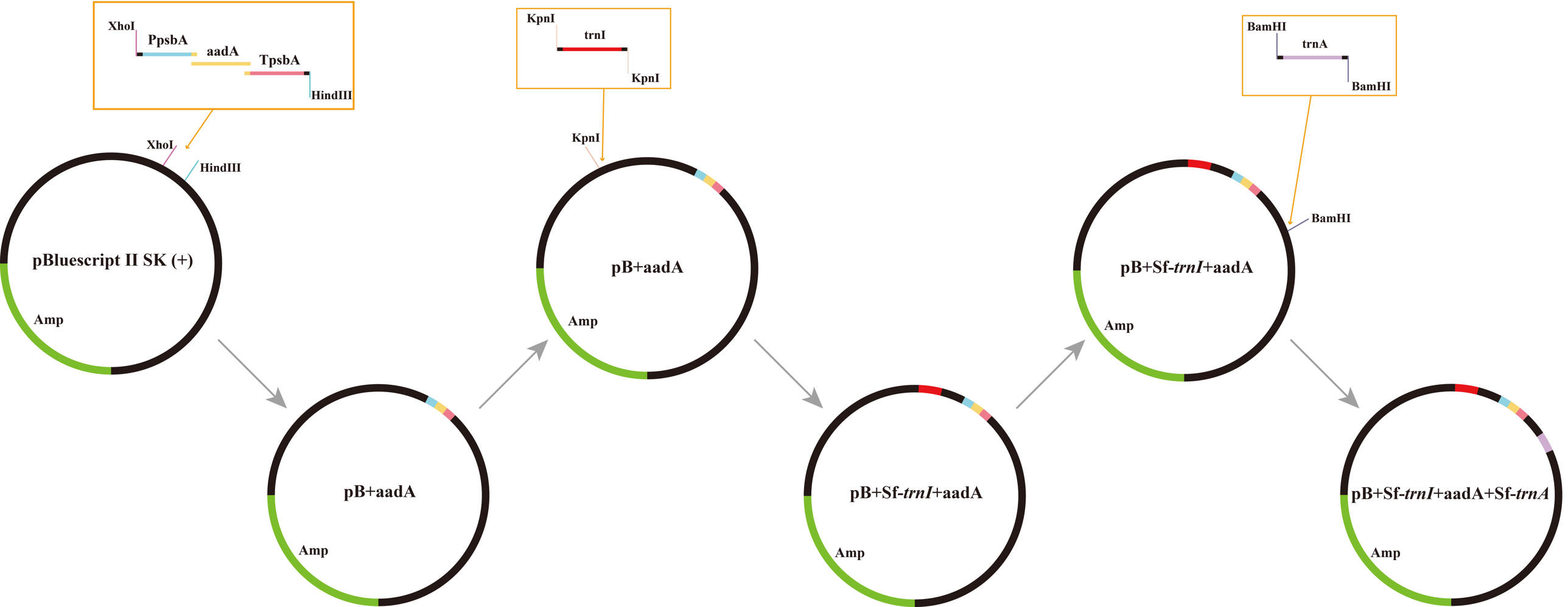

Supplement: Supplementary file 1 [file Image_1.JPEG]

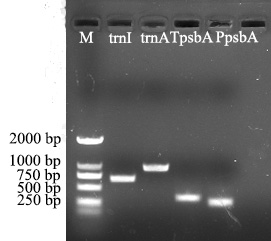

Supplement: Supplementary file 2 [file Image_2.JPEG]
