# Supplementary material for: The chloroplast genome of Salix floderusii and characterization of chloroplast regulatory elements
Source: Front Plant Sci. 2022 Aug 26;13:987443. doi: 10.3389/fpls.2022.987443 (PMC9459086; doi:10.3389/fpls.2022.987443)
Supplement: Supplementary file 3 [file Table_1.docx]

| **Species** | **GeneBank** | **Species** | **GeneBank** |
| --- | --- | --- | --- |
| *Populus davidiana* | NC_032717 | *Salix magnifica* | NC_037424 |
| *Salix alba* | MW435414 | *Salix maizhokunggarensis* | MN952983 |
| *Salix argyracea* | NC_056250 | *Salix matsudana* | NC_059039 |
| *Salix babylonica* | KT449800 | *Salix minjiangensis* | NC_037425 |
| *Salix brachista* | NC_058984 | *Salix myrtilloides* | MW435441 |
| *Salix caesia* | MW435422 | *Salix oreinoma* | NC_035743 |
| *Salix caprea* | MW435424 | *Salix paraplesia* | NC_037426 |
| *Salix chaenomeloides* | NC_037422 | *Salix pentandra* | MW435443 |
| *Salix cheilophila* | MN495628 | *Salix psammophila* | NC_051969 |
| *Salix chienii* | MW969692 | *Salix purpurea* | NC_026722 |
| *Salix cinerea* | MW435426 | *Salix rehderiana* | NC_037427 |
| *Salix cupularis* | MZ365446 | *Salix rorida* | NC_037428 |
| *Salix dasyclados* | NC_056251 | *Salix sinopurpurea* | NC_054198 |
| *Salix dunnii* | CM027679 | *Salix suchowensis* | NC_026462 |
| *Salix fragilis* | MW435430 | *Salix taoensis* | NC_037429 |
| *Salix gordejevii* | NC_058001 | *Salix tetrasperma* | NC_035744 |
| *Salix gracilistyla* | NC_043878 | *Salix triandra* | MW435451 |
| *Salix hastata* | MW435433 | *Salix triandroides* | MW929215 |
| *Salix hypoleuca* | NC_037423 | *Salix variegata* | NC_057289 |
| *Salix integra* | NC_056253 | *Salix viminalis* | MN117720 |
| *Salix koriyanagi* | MK120982 | *Salix repens* | MW435447 |
| *Salix linearistipularis* | MZ018223 | *Salix wilsonii* | NC_053549 |
| *Salix lindleyana* | NC_063129 |  |  |

**Supplementary Table 1**. Details of 45 chloroplast genome from 44 *Salix* genus species and *Populus davidiana* were downloaded from NCBI.
