# Supplementary material for: The chloroplast genome of Salix floderusii and characterization of chloroplast regulatory elements
Source: Front Plant Sci. 2022 Aug 26;13:987443. doi: 10.3389/fpls.2022.987443 (PMC9459086; doi:10.3389/fpls.2022.987443)
Supplement: Supplementary file 4 [file Table_2.docx]

**Supplementary Table 2.** Primers for all PCR.

| **Primer** | **Sequence (5’ to 3’)** |
| --- | --- |
| Sf-*PpsbA* F | aatggtaccgggccccccCCTAAATTTACAAATTACCAAG |
| Sf-*PpsbA* R | atcaccgcttcccccatGACTTTTGTAAATGGATAAGAC |
| aadA F | ATGGGGGAAGCGGTGATCGCCGA |
| aadA R | TTATTTGCCGACTACCTTGGTGATCTCGCC |
| Sf-*TpsbA* F | gtagtcggcaaataaGGTAAAATTTTGGTTTATTTAATC |
| Sf-*TpsbA* R | caggaattcgatatcCTATAACCATTCTAACTAATAT |
| Sf-*trnI* F | ctatagggcgaattgACGTATCTTCACAGACCAAGAAC |
| Sf-*trnI* R | taaatttaggggggggcccATTCTTTTCTTTGGCGCAGCTGGG |
| Sf-*trnA* F | aattcctgcagcccgggACATGCTCCACTTGGCTCGGGG |
| Sf-*trnA* R | cgctctagaactagtTACCGACTAAGCTCACGAGTTG |
