# Supplementary material for: The chloroplast genome of Salix floderusii and characterization of chloroplast regulatory elements
Source: Front Plant Sci. 2022 Aug 26;13:987443. doi: 10.3389/fpls.2022.987443 (PMC9459086; doi:10.3389/fpls.2022.987443)
Supplement: Supplementary file 5 [file Table_3.docx]

**Supplementary Table 3.** The number of all types of SSR in *S. floderusii*, *S. caprea* and *S. cinerea.*

| Species | Repeats | Amount |
| --- | --- | --- |
| *S. floderusii* | A/T | 188 |
|  | C/G | 12 |
|  | AG/CT | 18 |
|  | AT/AT | 37 |
|  | AAT/ATT | 1 |
|  | AAAC/GTTT | 1 |
|  | AAAG/CTTT | 2 |
|  | AAAT/ATTT | 2 |
|  | AATG/ATTC | 2 |
|  | AATT/AATT | 3 |
|  | AGAT/ATCT | 1 |
|  | AATAG/ATTCT | 1 |
| *S. caprea* | A/T | 186 |
|  | C/G | 12 |
|  | AG/CT | 18 |
|  | AT/AT | 37 |
|  | AAT/ATT | 1 |
|  | AAAC/GTTT | 1 |
|  | AAAG/CTTT | 2 |
|  | AAAT/ATTT | 2 |
|  | AATG/ATTC | 2 |
|  | AATT/AATT | 3 |
|  | AGAT/ATCT | 1 |
|  | AATAG/ATTCT | 1 |
| *S. cinerea* | A/T | 186 |
|  | C/G | 12 |
|  | AG/CT | 18 |
|  | AT/AT | 37 |
|  | AAT/ATT | 1 |
|  | AAAC/GTTT | 1 |
|  | AAAG/CTTT | 2 |
|  | AAAT/ATTT | 2 |
|  | AATG/ATTC | 2 |
|  | AATT/AATT | 3 |
|  | AGAT/ATCT | 1 |
|  | AATAG/ATTCT | 1 |
