# Supplementary material for: The chloroplast genome of Salix floderusii and characterization of chloroplast regulatory elements
Source: Front Plant Sci. 2022 Aug 26;13:987443. doi: 10.3389/fpls.2022.987443 (PMC9459086; doi:10.3389/fpls.2022.987443)
Supplement: Supplementary file 7 [file Table_5.docx]

**Supplementary Table 5.** Number of codons per amino acid of *S. floderusii.*

| AA | Codon | Amount |
| --- | --- | --- |
| Ala | GCU | 624 |
| Ala | GCC | 205 |
| Ala | GCA | 390 |
| Ala | GCG | 144 |
| Arg | CGU | 334 |
| Arg | CGC | 109 |
| Arg | CGA | 368 |
| Arg | CGG | 106 |
| Arg | AGA | 491 |
| Arg | AGG | 159 |
| Asn | AAU | 1017 |
| Asn | AAC | 311 |
| Asp | GAU | 852 |
| Asp | GAC | 229 |
| Cys | UGU | 214 |
| Cys | UGC | 92 |
| Gln | CAA | 727 |
| Gln | CAG | 214 |
| Glu | GAA | 1060 |
| Glu | GAG | 354 |
| Gly | GGU | 561 |
| Gly | GGC | 193 |
| Gly | GGA | 720 |
| Gly | GGG | 320 |
| His | CAU | 488 |
| His | CAC | 154 |
| Ile | AUU | 1149 |
| Ile | AUC | 441 |
| Ile | AUA | 730 |
| Leu | UUA | 908 |
| Leu | UUG | 578 |
| Leu | CUU | 598 |
| Leu | CUC | 195 |
| Leu | CUA | 406 |
| Leu | CUG | 185 |
| Lys | AAA | 1080 |
| Lys | AAG | 397 |
| Met | AUG | 639 |
| Phe | UUU | 1019 |
| Phe | UUC | 533 |
| Pro | CCU | 427 |
| Pro | CCC | 203 |
| Pro | CCA | 308 |
| Pro | CCG | 146 |
| Ser | UCU | 585 |
| Ser | UCC | 336 |
| Ser | UCA | 420 |
| Ser | UCG | 191 |
| Ser | AGU | 416 |
| Ser | AGC | 132 |
| Thr | ACU | 533 |
| Thr | ACC | 243 |
| Thr | ACA | 432 |
| Thr | ACG | 133 |
| Trp | UGG | 465 |
| Tyr | UAU | 797 |
| Tyr | UAC | 174 |
| Val | GUU | 498 |
| Val | GUC | 174 |
| Val | GUA | 542 |
| Val | GUG | 205 |
| TER | UAA | 44 |
| TER | UAG | 22 |
| TER | UGA | 20 |
